# High-frequency dynamics of evanescently-coupled nanowire lasers

**DOI:** 10.1038/s41598-019-42526-x

**Published:** 2019-04-16

**Authors:** M. J. Adams, D. Jevtics, M. J. Strain, I. D. Henning, A. Hurtado

**Affiliations:** 1 School of Computer Science and Electronic Engineering, University of Essex, Wivenhoe Park, Colchester, CO4 3SQ UK; 2 Institute of Photonics, SUPA Department of Physics, University of Strathclyde, TIC Centre, 99 George Street, Glasgow, G1 1RD UK

## Abstract

We analyse the dynamics and conditions for stability in an array of two laterally-coupled nanowire lasers in terms of their separation, difference in resonant frequencies and pumping rate under conditions of weak coupling. We find that the regions of stability are very small and are found close to zero frequency offset between the lasers. Outside these regions various forms of instability including periodic oscillation, chaos and complex dynamics are predicted. Importantly, the analysis of the frequency of periodic oscillations for realistic laser separations and pumping yields values of order 100 GHz thus underlining the significant potential of nanowire laser arrays for ultra-high frequency on-chip systems with very low foot-print and energy requirements.

## Introduction

Since the first reports in 2001^1^, research onto semiconductor nanowire (NW) lasers has blossomed into an important activity with promise of widespread applications in the future (for a recent review, see^2^). However, significant developments are needed before these applications can be realised. Among the most important of these is finding efficient techniques for electrical excitation, since at present most nanolasers are optically pumped. In addition progress is also required in establishing reliability and reproducibility. Another aspect that will be key to many applications is the requirement for integration with other nanophotonic components such as waveguides, modulators, photodetectors, Light Emitting Diodes (LEDs), etc. Significant achievements have been reported on arrays of solar cells based on nanowires^3^ and nanopillars^4^, nevertheless as yet there has been little progress on forming arrays of semiconductor nanowire lasers. While there have been recent reports on nanolaser arrays using other approaches^5–9^, e.g. photonic crystals^5^, coupled plasmonic structures^6^, perovskite nanolaser arrays^7^, individual waved nanoribbons^8^, coupled metallic nanodisks^9^, to name but a few, these realisations rely on complex fabrication stages and material combinations which can ultimately limit the flexibility in device design required for the development of coupled nanolaser arrays. In this respect, recent developments in hybrid nanofabrication based on nanoscale transfer printing techniques^10–13^ can offer new routes to integrate semiconductor nanowire (NW) lasers based on traditional III–V materials (e.g. InP, GaAs, etc) into arrays and even more complex nanophotonic circuits. Nanoscale transfer printing is a highly precise pick-and-place technique which uses polymer micro-stamps to controllably capture individually-selected semiconductor NW lasers from their original substrate for their subsequent release at desired locations in targeted surfaces with very high positioning accuracy^10–12^. Hence, by simply iterating this transfer printing processes, two selected NW lasers (or more than two if required) can be transfer-printed onto a desired surface with controlled (sub-micrometric) separation between them to form fully controlled arrays of two (or more) laterally-coupled NW lasers. Therefore, nanoscale transfer printing processes offer great promise for the fabrication of coupled nanolaser arrays with great control on the number and specific locations of the individual nanolasers and choice of substrates (e.g. polymers, silicon, metals, mechanically flexible substrates, etc^10–12^) or nanophotonic platforms (e.g. waveguides, nanoantennas, patterned substrates etc.^11,13,14^) on which these can be assembled. It is therefore timely to explore the behaviour of arrays of coupled NW lasers and so the present contribution focuses on the dynamics of evanescently-coupled nanolasers and their potential for very high frequency operation with extremely reduced footprint.

Coupled laser dynamics are attracting increased research interest, as highlighted by recent work on coupled photonic crystal vertical cavity surface-emitting lasers (PC-VCSELs)^15–17^. These allow precise control of the coupling between adjacent lasers by changing the diameter of the hole in the ‘photonic barrier’ between emitters^15^. Pairs of evanescently-coupled PC-VCSELs have been used to demonstrate spontaneous mirror-symmetry breaking through a pitchfork bifurcation^16^, and modal switching between in-phase and out-of-phase modes^17^. Coupling strength in these demonstrations is measured by the product of coupling rate $\kappa$ and photon lifetime ${\tau }_{p}$. Strong coupling is typically characterised by $\kappa {\tau }_{p} > 1$ (as in, e.g.^17^ with values of 8 and 12). Recent demonstrations of hybrid nanolaser integration technologies^10–13^ open up a complementary technology to assemble ultrasmall coupled laser systems with accurate nanoscale separations and controlled coupling strengths between NWs in the weakly-coupled regime characterised by $\kappa {\tau }_{p} < 1$. Until now, there has been little research reported on evanescent coupling of NW devices. An experimental study reported on the dependence of coupling efficiency on wavelength, angle, and core-diameter in NW evanescent wave coupling used a tapered silica fibre to couple light into a poly(trimethylene tereph-thalate) (PTT) nanowire^18^. Evanescent coupling between two parallel NW passive circular waveguides has been studied using finite difference time domain (FDTD) modelling for silica, tellurite and silicon as typical materials^19^, and an analysis of the influence of the refractive index of the ambient medium on coupling between microfibers or nanofibers was reported using conventional coupled mode theory^20^. In the case of NW laser integration, to date only limited cavity mode selection has been demonstrated using either coarse micromanipulation^21^ or complex post-processing of Gallium Nitride (GaN) NW devices^22^. In these works^21,22^, single-mode lasing via mode selection mechanisms were reported, even though the individual NWs supported emission in multiple modes. In^21^ a pair of coupled GaN NW lasers with different lengths yielded single-mode emission via a mode selection mechanism through the Vernier effect, whereas in^22^ single mode emission was achieved from GaN cleaved-coupled NW cavities fabricated by cutting a long GaN NW into two devices of different dimensions, thereby yielding two Fabry-Perot cavities axially coupled through a nanometric air gap. Until now, studies on coupled NW laser systems have therefore been scarce given the traditional difficulties to fabricate such systems. However, nanoscale integration technologies^10–13^ combined with recent experimental developments in the dynamical operation of evanescently-coupled lasers (e.g. PC-VCSELs^15–17^) opens up a new research avenue in coupled NW laser dynamics. The present contribution focuses on the stability, dynamics and prospects for very high frequency oscillation in evanescently-coupled pairs of NW lasers operated in the weak coupling regime. We determine the bifurcations separating regions of stable and unstable dynamics, using accurate yet simple approximations^23^, to show that weakly-coupled NW laser arrays can be expected to exhibit a wide range of behaviour including periodicity with high frequencies, complex dynamics and chaos.

## Results

**Figure 1:**
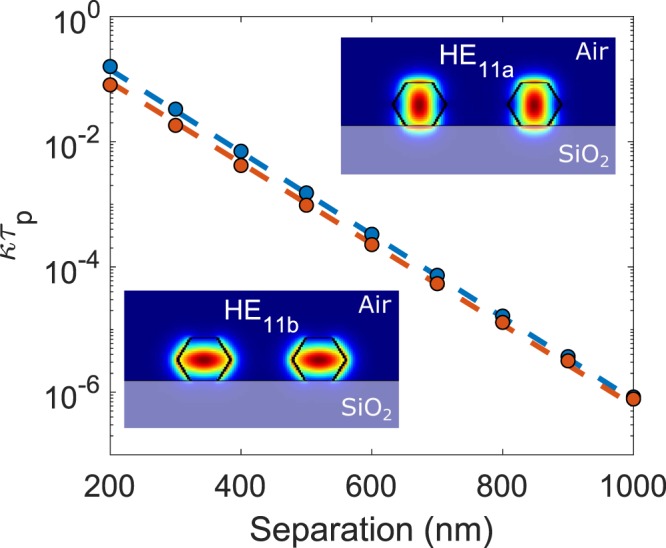
Relationship between $\kappa {\tau }_{p}$ and the separation between NWs for the HE_11*a*_ (in blue) and HE_11*b*_ (in orange) modes. Comparison of results obtained from FDE simulations (solid circles) with those from equation (1) (broken lines). The inset shows the intensity profiles of the two modes supported by the investigated coupled nanowire laser structure.

The coupling rate $\kappa$ can be calculated from the propagation constants *β*_1_, *β*_2_ of the symmetric and antisymmetric normal modes of the coupled NW laser structure using the relation $\kappa =({\beta }_{1}-{\beta }_{2})c$/$(2n)$, where *c* is the speed of light and *n* is the refractive index of the active medium. We have calculated $\kappa$ for Indium Phosphide (InP) NW lasers on a silica substrate (see Fig. 1). We must note here that the reasons behind the choice of InP NW lasers in this study are two-fold; firstly, although reported only very recently^24^, there are already significant publications focusing on their lasing properties and physical parameter values, information essential for the present study^24,25^. Secondly, InP NW lasers have already been succesfully used in combination with nanoscale transfer printing techniques to form large arrays and complex nanophotonic systems (e.g. waveguides, nanoantennas)^11–13^. Therefore, InP NW lasers are in our view one of the most promising candidates for early realisations of laterally-coupled NW lasers, offering assembly on different substrates and enabled by new hybrid nanofabrication protocols. The InP NW lasers were configured with the following parameter values: InP refractive index *n* = 3.4, substrate refractive index 1.5, NW hexagonal core diameter 2*a* = 260 nm and wavelength *λ* = 880 nm^25^ for a range of edge-to-edge separations, 2*d*, from 200 nm to 1000 nm using Finite Difference Eigenmode (FDE) software. The two InP NWs are set with equal lengths of length 5 *μ*m (here we assume the lasers are identical although small and unavoidable differences between a pair of NW lasers will exist in practice). For these parameter values only the fundamental HE_11_ mode is guided in each laser (following the notation used for circular cylindrical waveguides). The two polarisations of this mode are distinguished by the notation HE_11*a*_ and HE_11*b*_, with the subscript ‘a’ referring to the case with the higher effective index. The intensity profiles for the HE_11*a*_ and HE_11*b*_ modes of the coupled InP NW laser system investigated in this work are shown in the insets in Fig. 1.

In order to determine the bifurcations defining regions of stability/instability we require a very fine mesh in separation. It is not practical to use FDE modelling at each mesh point and hence we fit the computed results to analytical approximations of the form^23^:

1 $$\kappa =C{\exp }(\,-2w\frac{d}{a})$$

where $w=\frac{2\pi a}{\lambda }\sqrt{{n}_{e}^{2}-{n}_{clad}^{2}}$, with *n*_*clad*_ being here the refractive index of air (*n*_*clad*_ = 1) and *n*_*e*_ the effective index of each individual NW waveguide mode found from the FDE simulations. The constant *C* is found from fitting to the simulated variation of coupling rate $\kappa$ with laser separation 2*d*. Numerical results for *w* and *C* for coupling between identical modes are shown for the HE_11*a*_ and HE_11*b*_ modes in Table 1 (these are the only two modes that propagate for this NW core diameter). The Table also shows the values of the photon lifetime for each mode, found from ${\tau }_{p}={({v}_{g}{\rm{\Gamma }}{g}_{th})}^{-1}$, where *v*_*g*_ is the group velocity, ${\rm{\Gamma }}$ is the mode confinement factor, and *g*_*th*_ is the gain per unit length at threshold. Values of the latter two quantities are found from simulations in^25^ for InP NW lasers of length 5 *μ*m.

**Table 1:** Numerical values of parameters.

Mode	*w*	*C* (ps^−1^)	*τ*_*p*_ (ps)
HE11a	1.9733	45	0.0656
HE11b	1.9321	23	0.0757

Results of the product $\kappa {\tau }_{p}$ versus 2*d* calculated using these parameter values in equation (1) are compared with results from FDE simulations in Fig. 1. It is seen that equation (1) is an excellent approximation for the HE_11*a*_ and HE_11*b*_ modes. The normalised rate equations that describe the coupled lasers are^23^:

2 $$\frac{d{Y}_{A}}{dt}=\frac{1}{2{\tau }_{p}}\,({M}_{A}-1)\,{Y}_{A}-\kappa {Y}_{B}\,\sin (\varphi )$$

3 $$\frac{d{Y}_{B}}{dt}=\frac{1}{2{\tau }_{p}}\,({M}_{B}-1)\,{Y}_{B}+\kappa {Y}_{A}\,\sin (\varphi )$$

4 $$\frac{d\varphi }{dt}=\frac{{\alpha }_{H}}{2{\tau }_{p}}\,({M}_{A}-{M}_{B})-\frac{{\rm{\Delta }}f}{2\pi }+[\frac{{Y}_{A}}{{Y}_{B}}\,\cos (\varphi )-\frac{{Y}_{B}}{{Y}_{A}}\,\cos (\varphi )]$$

5 $$\frac{d{M}_{A,B}}{dt}=\frac{1}{{\tau }_{n}}\,[Q-{M}_{A,B}\,(1+{Y}_{A,B}^{2})]$$

where *Y*_*A*_, *Y*_*B*_ are the normalised fields and *M*_*A*_, *M*_*B*_ are the normalised carrier densities in guides A, B, respectively, $\varphi$ is the phase difference between the fields in B and A, Δ*f* is the frequency detuning between the cavity resonances of lasers B and A, ${\tau }_{n}$ is the carrier lifetime, *α*_*H*_ is the linewidth enhancement factor and Q is the normalised pumping rate, assumed the same in lasers A and B. With the aid of the approximation from (1), it is possible to study the dynamics of coupled NW lasers using the theory in^23^. In order for the approximations in^23^ for the bifurcations to hold, the condition $\kappa {\tau }_{p}\ll 1$ must be satisfied. From Fig. 1 this means attention is limited to the region 2*d* > 300 nm for the HE_11*a*_ and HE_11*b*_ modes. For a frequency offset Δ*f* between nanolasers A and B, the stability conditions are given by^23^:

6 $${\rm{\Delta }}f < \frac{{\alpha }_{H}\kappa }{\pi }$$

7 $${\rm{\Delta }}f > \frac{1}{2\pi }\sqrt{{(2{\alpha }_{H}\kappa )}^{2}-{(\frac{Q}{{\tau }_{n}})}^{2}}$$

Conditions (6) and (7) correspond to the saddle-node and Hopf bifurcations, respectively. It is important to note here that the offset in resonant frequency between the two nanolasers (Δ*f*) is the result of random unavoidable differences in the nanolasers’ cavity length during fabrication (e.g. a change in cavity length of just 0.1 nm will yield a frequency offset of 5 GHz) and not controllable frequency differences achieved by precisely tuning the cavity length (as is the case for example in frequency comb lasers).

**Figure 2:**
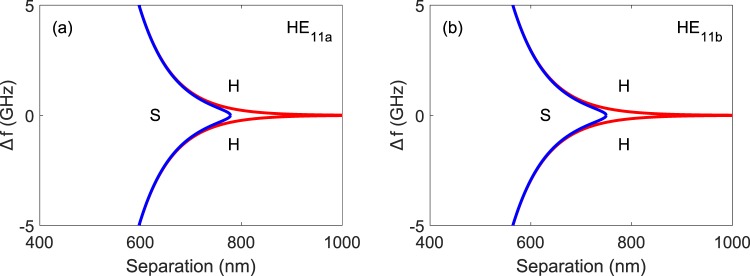
Saddle-node (S) and Hopf (H) bifurcations calculated from equations (6) and (7) for the HE_11*a*_ (**a**) and HE_11*b*_ (**b**) modes of the coupled InP NW laser system under analysis for *α*_*H*_ = 3, ${\tau }_{n}$ = 1 ns and Q = 2.

Figure 2 shows the bifurcations calculated for HE_11*a*_ and HE_11*b*_ modes using equations (6) and (7) with *α*_*H*_ = 3, ${\tau }_{n}$ = 1 ns and *Q* = 2. The regions of stability where the two lasers are phase-locked in the antiphase normal mode lie between these bifurcations. Outside these regions various forms of instability including periodic oscillation, chaos and complex dynamics are found^26^. The regions of stable locked behaviour are very small, mainly close to zero frequency offset between the two NW lasers. In practice this would imply the initial selection of devices which have similar lasing characteristics and resonance frequencies. In this connection a recent report describes a statistical technique for large-scale characterisation of nanowire lasers^27^. This could be used in principle to select devices with the required properties. Following this, any such chosen pair which would be pumped together would tend to track, thus maintaining a constant frequency difference.

From equation (7) it is clear that the minimum separation for stable phase-locked operation occurs for zero frequency offset and is given by:

8 $$Q=2{\tau }_{n}{\alpha }_{H}\kappa $$

**Figure 3:**
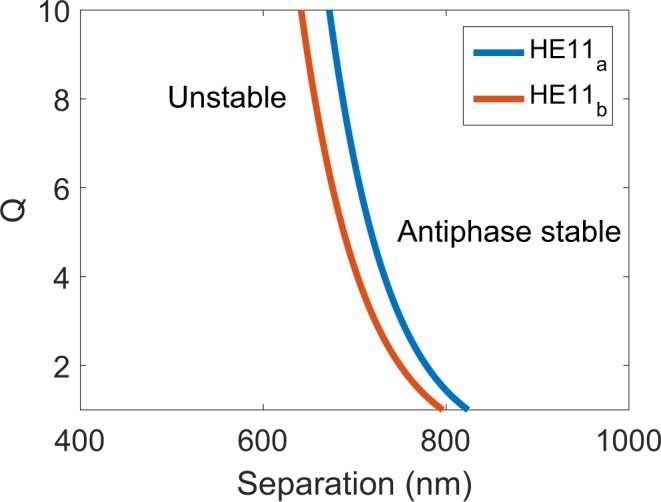
Stability boundaries for the HE_11*a*_ and HE_11*b*_ modes of the coupled InP NW laser system investigated in this work at zero frequency offset.

Figure 3 shows the variation of normalised pumping rate *Q* with laser separation 2*d* calculated for HE_11*a*_ and HE_11*b*_ modes from equation (8) for the same parameter values as in Fig. 2. The region of the stable antiphase normal mode lies to the right of the line for each mode. The normalised pumping *Q* is related to the actual pumping rate *P* by:

9 $$Q={C}_{Q}\,(\frac{P}{{P}_{th}}-1)+\frac{P}{{P}_{th}}$$

where *P*_*th*_ is the threshold value of *P* and ${C}_{Q}=\frac{{a}_{diff}}{{g}_{th}}{N}_{0}$, where *a*_*diff*_ is the differential gain and *N*_0_ is the transparency carrier concentration. These latter parameters are difficult to estimate for InP NW lasers, but for conventional lasers with, e.g. GaAs active regions, *a*_*diff*_ = 5 × 10^−16^ cm^2^ and *N*_0_ = 1 × 10^18^ cm^−3^. From^25^, *g*_*th*_ ~ 1870 cm^−1^ for the HE_11*a*_ mode and *g*_*th*_ ~ 1600 cm^−1^ for the HE_11*b*_ mode. Using these values gives *C*_*Q*_ ~ 0.27 for the HE_11*a*_ mode and *C*_*Q*_ ~ 0.31 for the HE_11*b*_ mode. It should be noted that values of these device parameters are subject to some uncertainty for nanowire lasers. The differential gain and transparency carrier concentration both influence the coefficient C_*Q*_ which affects the conversion from normalised pumping to the ratio of pump to threshold (although we do not attempt this conversion here). While the linewidth enhancement factor *α*_*H*_ is not expected to differ greatly from values found in other semiconductor lasers, the situation for the carrier and photon lifetimes is less clear. In the case of ${\tau }_{n}$ the effect of side-wall roughness and non-uniformity in nanowires might introduce non-radiative processes that shorten this lifetime. On the other hand, provided the ratio of pitch to hole diameter is not too large, the as-grown InP nanowires are hexagonal with smooth side walls and a planar top facet^25^, so we might not expect major reductions. As regards ${\tau }_{p}$, the values used here are derived, as detailed above, from calculated values of mode confinement factor, and threshold gain^25^. These short lifetimes are therefore the result of a relatively low facet reflectivity combined with a length of 5 *μ*m. Further discussion of the sensitivity of the results to the photon lifetime is given below.

In the regions of instability for zero frequency offset, a first-order approximation for the frequency *f* of periodic oscillations is given by^28^:

10 $$f=\frac{1}{2\pi }\sqrt{4{\kappa }^{2}+\frac{Q-1}{{\tau }_{n}{\tau }_{p}}}$$

**Figure 4:**
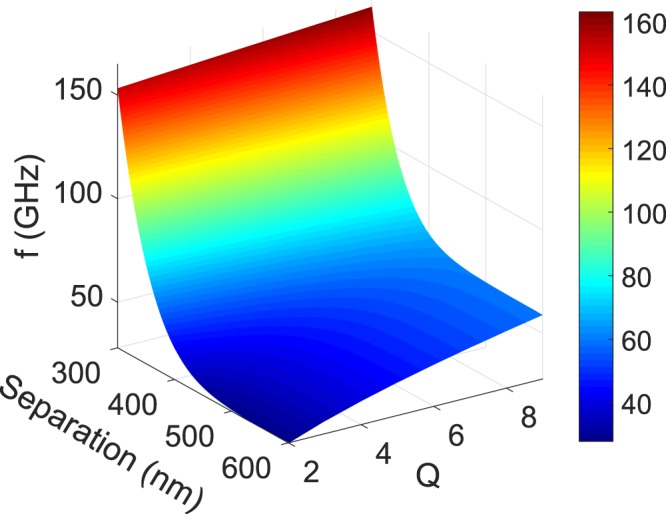
Frequency of periodic oscillations at zero frequency offset for the HE_11*a*_ mode.

The first term in the square root in (10) refers to the frequency of energy exchange (beating) between the two NW lasers, whilst the second term corresponds to the relaxation oscillation frequency. Figure 4 plots the frequency of periodic oscillations, *f*, versus the separation between NWs, 2*d*, and the normalised pumping, *Q*, for the HE_11*a*_ mode, for the same parameter values as above. It is seen that for lower separations very high frequencies of order 100 GHz are predicted.

Based on the results shown in Fig. 4, it is worth noting the potential for ultra-high-frequency modulation in these evanescently coupled NW lasers. If out-of-phase modulation is used, the modulation resonance frequency of laterally-coupled lasers is given by $\kappa$/*π*^29^, so the results of Fig. 4 for lower separations are a good guide to potential modulation speeds. Also, two-element arrays of photonic crystal VCSELs have been modulated at rates up to 37 GHz^30^. Modelling has shown that the phase between elements (equivalent to the frequency offset used here) and the injection ratio (equivalent to the coupling rate) are critical parameters that influence the modulation bandwidth. Further discussion of the effects of various parameters on the modulation frequencies of coupled pairs of lasers is given in^31^. It is important to see these new predictions of the high-speed potential of coupled nanowire lasers in the context of related work. First let us note that a simple rate equation approach has been used here, neglecting spontaneous emission coupled into the lasing modes. However, owing to the reduced mode volume in nanolasers the spontaneous and stimulated emission rates are modified^32^, leading to the prediction of ultrafast modulation speeds and a reduction of the turn-on delay time by as much as two orders of magnitude^33^. For a photonic crystal nanolaser operated at cryogenic temperatures between 7 K and 150 K, a measured delay time of 1.5 ps and direct modulation above 100 GHz have been reported^33^. Several schemes for achieving high speed modulation have been proposed, including coupled photonic crystal nanolasers with saturable absorbers^34,35^ optical injection into a nanolaser^36^ and mutually-coupled pairs of nanolasers^37^. However, experimental realisation of high modulation rates at room temperature has remained elusive (see^38^ for a recent review). Moreover in our rate equation analysis it is assumed that the cavity decay rate is slower than the polarization response rate. Since typical times for the latter are of order 10 fs, it is arguable that more sophisticated approaches, such as microscopic theory^39^ or a Bloch equation model^40^, are needed to model the temporal behaviour. However, since such other approaches require additional parameters which introduce further uncertainty, the simpler rate equation approach was considered adequate for an initial investigation (as has been the case for other pioneering studies of nanolaser modulation schemes^33,35–37^). In a similar spirit we note that our approach does not account for other effects (e.g. wavelength chirp, parasitics, heating,...) that limit high-speed modulation in practical devices. Future work will explore the benefits of implementing a more complex methodology.

Finally, it is also important to note here that although this work focuses on laterally-coupled InP NW lasers with emission at 880 nm, the results of the present study, including the ultra-high values of oscillation frequencies predicted, are extendable to other types of NW lasers based on different material systems. The latter include existing GaN or ZnO NW lasers emitting at UV and visible wavelengths (see^2^ and references therein). In addition research based on GaSb devices^41,42^ and GaAs–(In, Al)GaAs core–multishell NWs^43^ for example offer great promise to push the operating wavelengths of semiconductor NW lasers into the telecom windows of 1310 and 1550 nm. We note also that our work can easily be generalised to other nanowire types or geometries. In this context it has been shown that circular and hexagonal nanowires have the same behaviour if they have the same cross-sectional area^44^. However, for the purposes of integration, a hexagonal structure is more convenient as it gives larger area of contact to the substrate. Hence, our work clearly opens the way for nanolaser-enabled ultra-high frequency systems operating at diverse wavelength ranges for on-chip communications, spectroscopy and sensing functionalities.

## Discussion

To summarise, in this work we report a stability analysis of laterally-coupled pairs of nanowire lasers. We take as an example the case of InP devices on a silica substrate. We have determined the regions of stable and unstable behaviour in these nanoscale laser systems using simple but accurate approximations. Stability for the lowest-order modes, HE_11*a*_ and HE_11*b*_, is found to be confined to very small regions of small detuning between the resonant frequencies of the lasers. The frequency of sustained periodic oscillations in regions of instability is estimated to be in excess of 100 GHz for low but realistic device separations, thus indicating the potential for ultra-high frequency modulation beyond the limit of the relaxation oscillation frequency.

## Methods

We have modeled an array of two laterally-coupled Indium Phosphide (InP) NW lasers on a silica substrate for a range of (edge-to-edge) separations between devices, 2*d*, from 200 nm to 1000 nm. To do so we have used a combination of Finite Difference Eigenmode (FDE) simulations and analytical approximations^23^. The parameters for the InP NW lasers were as follows: InP refractive index *n* = 3.4, substrate refractive index 1.5, NW hexagonal core diameter 2*a* = 260 nm, NW length = 5 *μ*m and emission wavelength *λ* = 880 nm. For this choice of parameters the HE_11*a*_ and HE_11*b*_ modes are the only ones supported by the InP NWs. By means of the FDE simulations we obtain the effective indices of the normal modes of the full structure and hence the coupling rate between the guided modes of the NW lasers. The coupling rates are fitted to computed results for both supported modes obtained from analytical approximations based on coupled mode theory. This allows the use of the analytical model from^23^ to accurately determine the stability conditions and oscillation frequency of the investigated arrays of 2 laterally-coupled InP NW lasers. Results are obtained as a function of important system parameters including the frequency offset between devices, Δ*f*, separation between NWs, 2*d*, and normalised pumping rate, *Q*.

## Data Availability

All data underpinning this publication are openly available from the University of Strathclyde KnowledgeBase at 10.15129/3dff40d8-ec87-4743-a46c-a6e9b5e3d255.
